# Fabrication of Graphene/Molybdenum Disulfide Composites and Their Usage as Actuators for Electrochemical Sensors and Biosensors

**DOI:** 10.3390/molecules24183374

**Published:** 2019-09-17

**Authors:** Jiri Kudr, Vojtech Adam, Ondrej Zitka

**Affiliations:** 1 Department of Chemistry and Biochemistry, Mendel University in Brno, Zemedelska 1, Brno CZ-613 00, Czech Republic; kudr@mendelu.cz (J.K.); vojtech.adam@mendelu.cz (V.A.); 2 Central European Institute of Technology, Brno University of Technology, Brno CZ-616 00, Czech Republic

**Keywords:** 2D materials, bioanalysis, biomarker, carbon, electrode

## Abstract

From the rediscovery of graphene in 2004, the interest in layered graphene analogs has been exponentially growing through various fields of science. Due to their unique properties, novel two-dimensional family of materials and especially transition metal dichalcogenides are promising for development of advanced materials of unprecedented functions. Progress in 2D materials synthesis paved the way for the studies on their hybridization with other materials to create functional composites, whose electronic, physical or chemical properties can be engineered for special applications. In this review we focused on recent progress in graphene-based and MoS_2_ hybrid nanostructures. We summarized and discussed various fabrication approaches and mentioned different 2D and 3D structures of composite materials with emphasis on their advances for electroanalytical chemistry. The major part of this review provides a comprehensive overview of the application of graphene-based materials and MoS_2_ composites in the fields of electrochemical sensors and biosensors.

## 1. Introduction

In the last decade we were witnesses of a rapid development of materials science research. Inspired and motivated by the legendary Feynman’s lecture, many scientists are watching to “the bottom” and focusing on the unique physicochemical and mechanical properties of nanomaterials. In the 1960’s, a simple scotch tape technique was used to mechanically exfoliate bulk MoS_2_ to a few-layers state; this also enabled the isolation of other layered materials decades later, including graphene [1,2,3]. After pristine graphene isolation in 2004, burgeoning research in the field of layered 2D materials began [2]. Nowadays, we can use several top-down and bottom-up synthetic procedures to obtain materials with exact numbers of layers and specific properties. In addition, stacking of layered materials on each other due to van der Waals forces can be used to engineer heterostructured solids with unprecedented properties [4].

2D layered materials represent the thinnest (atomically thin) unsupported crystalline solids without dangling bonds thus providing superior intralayer transport of light, heat, spin and charge [5]. Graphene, a single layer of carbon atoms bound in hexagonal honeycomb lattice, is a prominent member of the 2D layered materials group. It possesses several extraordinary properties extensively described elsewhere [6]. Thevenot et al. described an electrochemical biosensor as an integrated receptor–transducer device which provides selective quantitative or semi-quantitative analytical information using a biological recognition element and an electrochemical transducer [7]. The integration of graphene-based materials with an electrochemical transducer provides several advantages, such as increased conductivity, electron transfer rate and/or increased surface-to-volume ratio of transducer thanks to graphene’s ultrathin structure [8,9,10,11]. Further, graphene’s ability to be functionalized with heteroatoms, various molecules or functional groups is part of the superior property of this unique material. The electronic and chemical properties of graphene-based materials are highly influenced by the content of oxygen functional groups. Graphene oxide (GO) and reduced graphene oxide (rGO), as oxygenated monolayers of carbon atoms are widely applied in bioelectronics and biosensors due to higher hydrophilicity (compared to graphene) and the presence of oxygen-containing groups, which enable broad possibilities for functionalization. It is worth noting that conductivity of graphene decreases with increasing oxygen content, thus a material of optimal C/O ratio is needed for individual applications.

Transition metal dichalcogenides (TMDs) are graphene analogs formed by stacking of sulfur-transition metal–sulfur sheets through van der Waals forces. As a consequence, individual layers can be exfoliated from bulk material to single-layer form. Molybdenum disulfide (MoS_2_) is a typical member of TMDs. It is composed of a stack of hexagonal layers of Mo atoms sandwiched between two layers of S atoms and possesses p-type semiconducting properties with poor electrical conductivity. MoS_2_ has two main advantages in electrochemical (bio)sensors. Firstly, it provides additional electrochemically active sites. Secondly, it can improve transducer properties by accommodating additional elements such as metal nanoparticles or biorecognition (e.g., antibodies, enzymes, aptamers) [12]. Similar to graphene, MoS_2_ possesses different affinities towards ssDNA and dsDNA [13]. Such behavior enables integration of MoS_2_ with broadly used aptamer technology. However, 2D MoS_2_ still suffers from several drawbacks. Restacking of 2D materials due high surface energy can decrease the amount of electrochemically active sites, and poor electrical conductivity limits its use as transduction enhancer. Therefore, the hybridization of 2D MoS_2_ with highly conductive materials such as graphene can help to overcome mentioned drawbacks and can result in materials with fascinating electroanalytical properties.

Several reviews showing the use of MoS_2_ or the group of TMDs in the field of electrochemical (bio)sensors have been published [14,15,16,17], however here we focus strictly on graphene-based composite materials with MoS_2_ (Gr/MoS_2_). We briefly summarize the most important synthetic procedures providing mentioned material. The vast majority of this review focuses on the electroanalytical performance of devices and different state-of-art electrochemical biosensing approaches benefiting from the unique properties of graphene/MoS_2_ composites.

## 2. Synthesis of Gr/2D MoS_2_ Composites

Many different methods have been developed for fabrication of Gr/MoS_2_ hybrid materials of exact properties such as thickness, morphology, number of electroactive sites. Desired properties of materials correspond with their application purposes and for this reason a significant part of this review is focused on Gr/MoS_2_ synthesis.

### 2.1. Hydrothermal and Solvothermal Synthesis

These methods are based on growth of MoS_2_ structures over graphene/GO/rGO support. MoS_2_ molecular precursors (e.g., Na_2_MoO_4_ and thioacetamide) are dissolved in aqueous (hydrothermal) or organic solvents (solvothermal synthesis) and they are thermally treated in stainless steel reactors for an exact time and at a specific temperature above boiling point of solvent. Products of various properties can be obtained by optimization of heating temperature, reaction time and presence of additional compounds such as surfactants. Advantages of these methods are high yields, simple procedures and relatively low cost of equipment. However, the biggest drawback is low control of the synthesis process.

**Figure 1:**
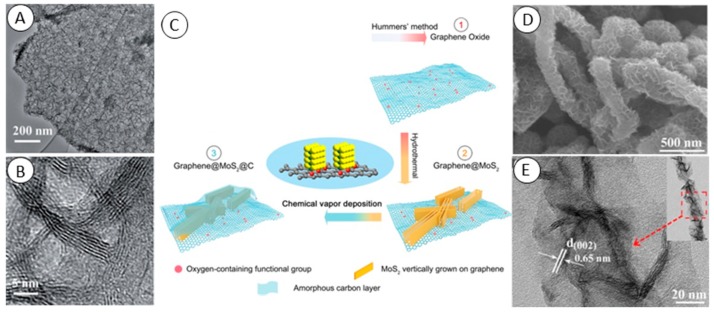
(**A**) TEM images and (**B**) HRTEM images of vertically aligned exfolliated graphite and MoS_2_ composite. From Wang et al. [22]. (**C**) Schematic illustration of the formation of the graphene/MoS_2_/amorphous carbon composite. From Teng et al. [23]. (**D**) FE-SEM and (**E**) TEM images of the MoS_2_ and carbon nanofiber composite. The insert shows the TEM image of a single MoS_2_ and carbon nanofiber nanostructure. From Li et al. [29].

A few-layer MoS_2_ hybrid with GO was synthesized by Yu et al. [18]. They used N-methylimidazole water-soluble pillar [5] arene as a surfactant to improve distribution of MoS_2_ sheets on commercial GO. They heated a solution containing 7 mg∙mL^−1^ of GO, 1.5 mmol Na_2_MoO_4_, 7.5 mmol L-cysteine dissolved in 40 mL deionized water at 240 °C for 24 h. The freeze-dried product was annealed in a tube furnace at 500 °C for 2 h in flow of 10% hydrogen in nitrogen. This approach provided laterally (in-plane) stacked MoS_2_ on Gr/GO/rGO substrates as reported in other publications with alternative surfactants or without surfactant [19,20,21]. On the contrary, without surfactant, MoS_2_ vertically stacked on electrochemically exfoliated graphite (EG) as a substrate was reported by Wang et al. [22]. They mixed EG with (NH_4_)_2_MoS_4_ in weight ratios from 1:1 to 1:13 in the presence of hydrazine and treated these mixtures in stainless steel reactors at 200 °C for 15 h and obtained Gr/MoS_2_ with 95 wt% MoS_2_ content. The product of this reported synthesis can be seen in Figure 1A,B. Hydrothermal growth of MoS_2_ on GO in the presence of polyvinylpyrrolidone (PVP) and oxalate dihydrate was reported by Teng et al. [23]. As a MoS_2_ precursors, they used ammonium paramolybdate (NH_4_)_6_Mo_7_O_24_ and thiourea and heated their mixture with GO at 180 °C for 12 h in 100 mL autoclave. Then the material was annealed at 800 °C in argon atmosphere to obtain rGO/MoS_2_. Finally, they covered rGO/MoS_2_ with amorphous carbon using chemical vapor deposition (CVD) to enhance charge transfer through composite (Figure 1C). Hydrothermal and solvothermal methods are able to fabricate graphene and MoS_2_ hybrid materials with various 2D and 3D structures. As reported Sun et al., just by changing the rGO amount in the reaction, 3D assembly of MoS_2_ can be tuned from nanoflowers to crosslinked nanosheets laterally stacked on GO [24]. They used mixed solvothermal synthesis in an ethanol and octylamine mixture. Mixed solvents of deionized water and dimethylformamide (DMF) in a 1:2 ratio were used by Zhao et al. [25]. They observed the effect of NH_3_·H_2_O concentration in the reaction reactor on the final product and compared the results with solution with pH adjusted with NaOH. When NH_3_·H_2_O was added to reaction mixture, the 3D porous framework constructed by interconnected lamellar nanosheets of Gr/MoS_2_ hydrogel became more homogeneous with pore sizes from hundreds of nanometers to micrometers. In addition, the presence of NH_3_·H_2_O resulted in graphene N-doping. Solvothermal and hydrothermal approaches were also used to modify the 1D carbon fiber, carbon nanotubes or macroscopic carbon paper [26,27,28,29]. MoS_2_ stacked on carbon nanofiber as was reported by Li et al. can be seen in Figure 1D,E.

### 2.2. Thermal and Chemical Reduction

**Figure 2:**
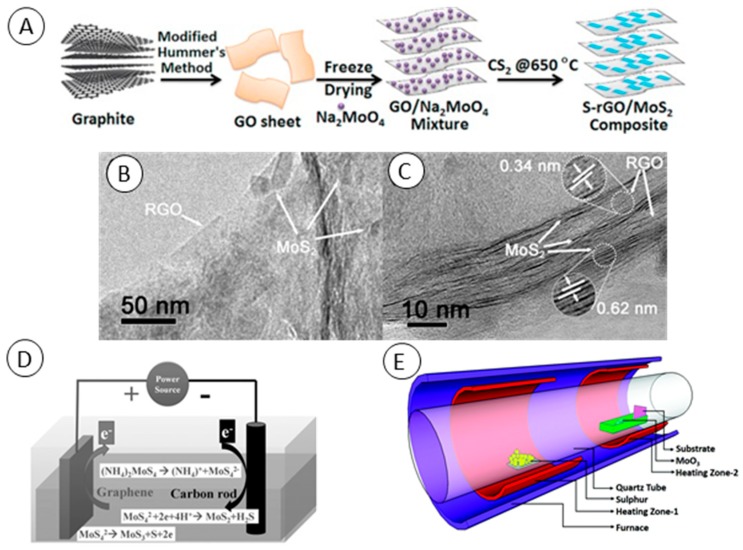
(**A**) Schematic representation showing synthesis of S-doped reduced graphene oxide (rGO)/MoS_2_ composite. From Wang et al. [32]. (**B**) Low-magnification and (**C**) high-magnification TEM images of rGO/MoS_2_ composite fabricated by one-pot microwave synthesis. From Li et al. [37]. (**D**) Schematic diagram of the chemical bath deposition of MoS_2_ on graphene as the anode and carbon rod as the cathode. From Wan et al. [41]. (**E**) Schematic of the two-zone chemical vapor deposition (CVD) furnace utilized for the synthesis of vertical MoS_2_ on graphene. From Gnanasekar et al. [44].

These methods of Gr/MoS_2_ synthesis are even more facile than hydrothermal and solvothermal ones. In case of thermal reduction, the thermal energy is used to convert MoS_2_ molecular precursors to MoS_2_ solid materials which are stuck on graphene-based support. In case of chemical reduction, chemical reductants are used to reduce MoS_2_ precursors to 2D MoS_2_. Although these methods can easily produce Gr/MoS_2_ in gram-scale, the main drawback is the control over synthesis process and that the properties of the product can hardly be predicted. The common procedure for Gr/MoS_2_ fabrication using thermal reduction is to dissolve the Mo salt with S source to create homogeneous solution which is subsequently dried and thermally treated at several hundred °C in inert atmosphere. Srivastava et al. fabricated MoS_2_ and graphene oxide composite by thermal exfoliation and reduction [30]. They ground ammonium thiomolybdate and graphite oxide and treated the powder for 6 h at 400 °C and finally for 15 min at 1200 °C under flow of nitrogen gas. Koroteev et al. fabricated Gr/MoS_2_ composite by impregnation of graphene with Mo-containing compound followed by thermal decomposition [31]. They dispersed graphene flakes in water–ethanol mixed solution with addition of ammonium thiomolybdate. Before thermal conversion (500–800 °C for 1 h) of MoS_3_ to MoS_2_ at vacuum, thiomolybdate was decomposed using HCl and dried at air atmosphere. Instead of thermal reduction in inert gas or vacuum, synthesis in the presence of CS_2_ results in S-doping of graphene-based materials (Figure 2A) [32]. Freeze-drying of graphene-based materials with MoS_2_ precursors provides benefits of sponge-like 3D products before thermal reduction [33]. As Jiang et al. mentioned, these 3D structures can help to prevent graphene or MoS_2_ restacking problems during synthesis and further processing [34]. They fabricated Gr/MoS_2_ of stable 3D structure using a combination of hydrothermal and chemical reduction methods. Firstly, they fabricated monolayer MoS_2_ and graphene oxide. They mixed GO and MoS_2_ in isopropanol/water solution where functional groups of GO attracted MoS_2_. The solution was hydrothermally treated to create porous 3D architecture. Subsequently the composite, or more precisely GO, was reduced using hydrazine. Ji et al. fabricated rGO/MoS_2_ by dry ball-milling of MoS_2_ and bulk GO at a ratio of 1:1 for 5 h [35]. The product was subsequently chemically reduced with hydrazine. Wang et al. used hydrazine to reduce (NH_4_)_2_MoS_4_ too. In addition, they used cetyltrimethylammonium bromide (CTAB) as cationic surfactant to promote interaction between negatively charged GO and MoS_4_^2−^ [36].

### 2.3. Microwave and Electrochemical Synthesis

Microwave synthesis is an effective method for Gr/MoS_2_ fabrication and represents an alternative to thermal methods. It can induce rapid decomposition of MoS_2_ precursors and hence lowers the energy consumption and does not need any chemical reductant. Effective one-pot microwave-assisted solvothermal synthesis was reported by Li et al. [37]. They dispersed liquid-exfoliated graphene (0.71 mg∙mL^−1^) in N-methylpyrrolidone with addition of 1-dodecanethiol, Na_2_MoO_4_·2H_2_O and thiourea. Such solution was heated in a specialized glass reactor using microwave at 200 °C for 12 h. Post-synthesis treatment in tube furnace at 800 °C for 2 h was used to remove sulfur residues and improve product crystallinity (composite can be seen in Figure 2B,C). Xiang et al. decorated chemical vapor deposited graphene foam with MoS_2_ nanoflowers using a microwave reactor [38]. More precisely, they irradiated an aqueous solution of Na_2_MoO_4_·2H_2_O, thioacetamide and graphene foam in glass tube and kept it at 180 °C for 12 h. Far shorter irradiation of the reaction mixture was reported by Li et al. [39]. They kept a solution of GO, phosphomolybdic acid hydrate and thioacetamide adjusted to pH 7 at 150 °C and 150 W for 10 min.

The electrochemical approach to fabricate Gr/MoS_2_ composites represents a low-cost and fast method without the use of toxic compounds. Further, it seems to be the most suitable approach for application in electrochemical (bio)sensors since they can be fabricated directly by the electrochemists who are enabled to optimize product properties by using deposition conditions. Fabrication of Gr/MoS_2_-modified electrode without the need of any post-fabrication treatment was reported by Li et al. [40]. Firstly, they deposited rGO layer on fluorine-doped tin oxide (FTO) by electrochemical reduction of GO from aqueous solution at −1.2 V. MoS_2_ nanoparticles were subsequently deposited on the conductive surface of rGO layer through electrochemical deposition of ammonium thiomolybdate in KCl electrolyte at −1.0 V for 5 min. Wan et al. reported constant current deposition of vertical MoS_2_ structures over a CVD-covered graphene electrode [41]. They used a two-electrode system and (NH_4_)_2_MoS_4_ as an electrolyte. Two-electron reduction occurred at the carbon rod cathode which was covered with MoS_2_. On the graphene anode a thin film of MoS_3_ was oxidatively electrodeposited (Figure 2D). A highly crystalline Gr/MoS_2_-covered anode was obtained by treating anode in quartz tube at 800 °C.

### 2.4. Chemical Vapor Deposition

CVD is an effective method of high quality Gr/MoS_2_ composite fabrication which provides excellent control over the fabrication process. In general, the substrate (often metal) is exposed in a vacuum chamber to gaseous precursors which react and/or decompose to create the desired deposited material, and carrier gas removes byproducts. CVD of graphene is a well-established technique, however it still faces many problems hampering the further use of graphene in fundamental research and practice [42]. In addition, it requires expensive equipment and the process needs to be stringently coordinated, thus it seems to be more suitable for FET-based (bio)sensors [43]. Fabrication of vertically aligned MoS_2_ nanosheets on graphene using CVD was reported by Gnanasecar et al. [44]. They fabricated graphene using atmospheric pressure CVD of acetylene on Cu foil, and the graphene was transferred onto a SiO_2_ wafer using poly(methyl methacrylate) (PMMA) film. Freestanding graphene on wafer was obtained by chemical etching of Cu and PMMA film. For MoS_2_ structures growing on graphene they used a home-made two-zone CVD reactor (Figure 2E). MoO_3_ and sulfur precursors were placed in separately controlled heating zones under the flow of Ar. The heating zone with MoO_3_ was ramped up to 650 °C (15 °C∙min^−1^), then slowly heated to 750 °C (2 °C∙min^−1^) and kept at 750 °C for 10 min. For sulfurization, the sulfur zone was rapidly heated to 200 °C (20 °C∙min^−1^). Biroju et al. used the mechanical transfer method of MoS_2_ on graphene [45]. They separately fabricated large area graphene and MoS_2_ using CVD and MoS_2_ on SiO_2_ wafer was uniformly pressed on graphene using a hydraulic pressurizer. Chen et al. fabricated MoS_x_ composite with CVD graphene by dispersing graphene in (NH_4_)_2_MoS_4_ DMF solution and treating it in a quartz tube furnace at 120 °C and 500 °C for 1 h [46].

### 2.5. Alternative Approaches

Some alternative approaches which cannot be placed within above-mentioned categories were developed. A very facile method for Gr/MoS_2_ fabrication was developed by Kumar et al. [47]. First, they prepared graphite oxide solution by continuous magnetic stirring and sonication of graphite oxide in ethanol. After addition of bulk MoS_2_ into graphite oxide solution, ethanol was evaporated. Dry powder was treated with microwave irradiation (800 W, 130 s) for complete exfoliation of graphite oxide. Simple mixing of GO solution with MoS_2_ nanoparticles and annealing at 400 °C was reported by Venkatesan et al. [48].

## 3. Applications of Gr/2D MoS_2_ Composites

Graphene-based materials and MoS_2_ composites possess specific properties depending on their morphology and structure. The properties are mostly influenced by number of MoS_2_ layers, graphene degree of oxidation, composite plane or 3D structure. Thus they represent versatile materials, which were recognized as promising for several fields of science. Among others, electrochemical sensors and biosensors can benefit from Gr/MoS_2_ advances by improved analytical performance such as higher sensitivity, increased analyte selectivity, better peak-to-peak separation and/or a broader linear dynamic range. In this chapter, electroanalytical devices benefiting from Gr/MoS_2_ properties are divided into sensors and biosensors applications, which directly reduce/oxidize analyte and which use biological recognition elements, respectively.

### 3.1. Gr/2D MoS_2_ Composites in Electrochemical Sensors

**Figure 3:**
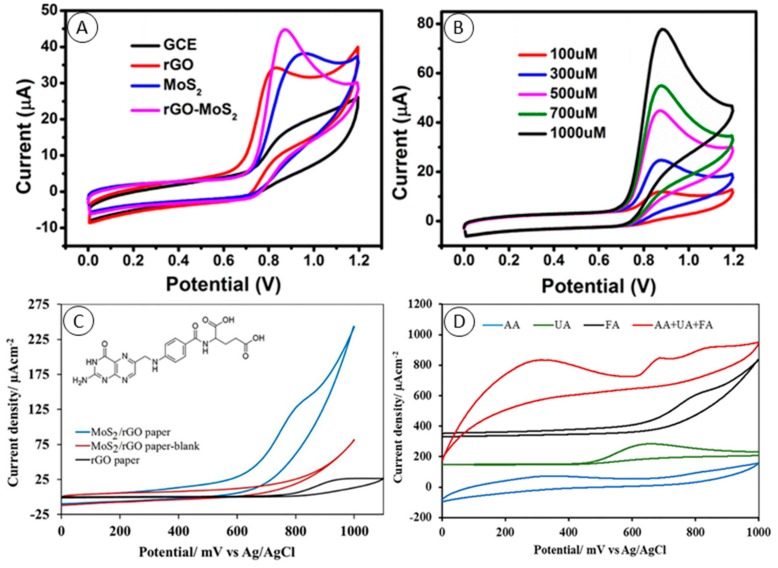
(**A**) Cyclic voltammetry CV curves of the glassy carbon electrode (GCE), GCE modified with rGO, MoS_2_ and rGO/MoS_2_ in 0.1 M PBS (pH = 7.0) with 500 μM nitrite. (**B**) CV curves of the GCE modified with rGO/MoS_2_ in 0.1 M PBS (pH = 7.0) under different concentrations of nitrite: 100, 300, 500, 700, and 1000 μM (scan rate: 50 mV∙s^−1^). Both from Hu et al. [55]. (**C**) CV curves of rGO (black) and rGO/MoS_2_ paper electrodes in 0.1 M PBS (pH 7.0) with (blue) and without (red) 2.0 mM folic acid (FA). Scan rate: 50 mV∙s^−1^. Inset: Structure of FA. (**D**) The CVs of the same concentrations of ascorbic acid (AA, blue line), uric acid (UA, green line) and FA (black line) and 1.0 mM AA, 0.5 mM UA and 0.5 mM FA containing solution (red line) at the rGO/MoS_2_ composite paper electrode in pH 7.0 PBS. Scan rate: 50 mV∙ s^−1^. Both from Kiransan et al. [59].

Electrochemistry represents a facile technique to detect small molecules with high sensitivity, low detection limits, reasonable cost and short time consumption. Uric acid (UA), dopamine (DA) and ascorbic acid (AA) are small biological molecules, which are presented in human physiological fluids and can help to determine human states, including several diseases. However their simultaneous determination is still challenging due to electrode fouling and their oxidation at almost the same potentials. The improvement of electrode sensitivity towards uric acid (UA), dopamine (DA) and ascorbic acid (AA) and differential pulse voltammetry (DPV) peak-to-peak separation via electrode modification with reduced graphene oxide composite with MoS_2_ was demonstrated by Xing et al. [49]. They showed that porous nanostructure of the composite increased specific surface of the electrode by nearly 4-fold compared with MoS_2_ modification, provided more active sites for target molecules adsorption and as a result improved electrode catalytic performance. The same conclusions were also made by Huang et al. in case of acetaminophen sensing in the presence of AA and DA with a similar electrode [50]. Direct oxidation of nitrite ions on conventional electrodes is still challenging due to the high overpotentials required. Thus, different modified electrodes were reported for nitrite sensing [51,52,53,54]. Gr/MoS_2_ can be used to decrease nitrite ions reduction potentials to +0.8 V vs. Ag/AgCl and increase sensitivity (Figure 3A,B) [55]. Three-dimensional nanostructured graphene, MoS_2_ flowers and multiwall carbon nanotubes (MWCNTs) composite was previously used for sensitive enzymeless determination of hydrogen peroxide [56]. An amperometric sensor based on modified glassy carbon electrode (GCE) showed excellent electrocatalytic activity towards reduction of H_2_O_2_, with a detection limit (LOD) of 0.83 μM, linear range of 5 µM–145 µM and sensitivity of 5.184 µA∙µM∙cm^−2^. Direct reduction of methyl parathion (its nitro functional group) was reported [57]. Methyl parathion belongs to the group of organophosphorus pesticides which generate concerns in food safety, water management and public health. Hydrothermally fabricated graphene MoS_2_ nanocomposite was used to modify GCE, and amperometry at −0.6 V vs. Ag/AgCl was used to compare the performance of bare, MoS_2_-modified and graphene-modified electrodes. A sensor was shown with parameters competitive to enzyme based detection—LOD 3.2 nM and linear dynamic range from 10 nM to 1.9 mM [58]. Good claimed selectivity of the sensor was ascribed to π-stacking of methyl parathion phenyl group on Gr/MoS_2_ composite, however discrimination was not possible between parathion and methyl parathion. Good performance of the sensor in spiked real fruit and vegetable samples was shown. A flexible electrochemical sensor of folic acid (FA) based on MoS_2_ nanosheet-modified rGO paper was designed by Kiransan et al. [59]. Their electrodes were fabricated by a two-step process. At first, the vacuum filtration of MoS_2_ and GO solution through polycarbonate membrane created a free-standing film of composite. Secondly, the film was reduced in HI solution for 1 h and then they cut the membrane to pieces of 5 × 10 mm. To obtain the best performance they optimized the ratio of GO and MoS_2_ in filtrated solution, finding that a 3:1 ratio was optimal. Oxidation of FA in 0.1 M PBS buffer (pH 7) took place at +0.73 mV vs. Ag/AgCl (Figure 3C). Amperometric detection resulted in LOD of 37 nM and the sensor showed good selectivity even in the serum and the presence of 1.0 mM AA and 0.5 mM UA (Figure 3D).

### 3.2. Gr/2D MoS_2_ Composites in Electrochemical Biosensors

**Figure 4:**
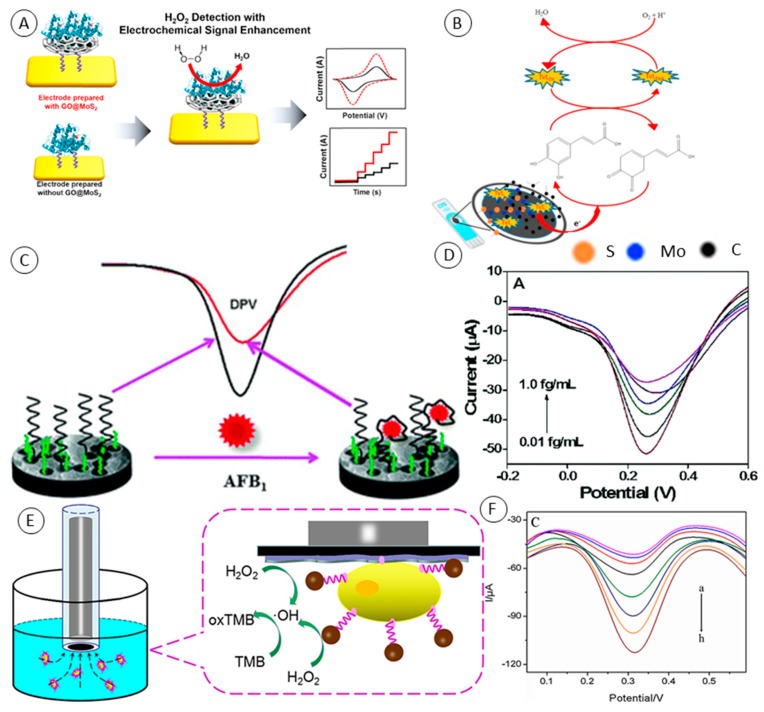
(**A**) Schematic of electrochemical biosensors composed of myoglobin (Mb) and of GO/MoS_2_ with electrochemical enhancement for H_2_O_2_ detection. From Yoon et al. [60]. (**B**) Schematic representation of construction and the detection principle of screen-printed carbon electrode modified with graphene quantum dots, MoS_2_ and laccase as a caffeic acid biosensor. From Vasilescu et al. [69]. (**C**) Schematic representation of the reduced graphene oxide/molybdenum disulfide/polyaniline nanocomposite-based electrochemical aptasensor for detection of aflatoxin B_1_ fabrication. (**D**) Differential pulse voltammetry (DPV) responses of the aptasensor after 20 min incubation with 0.0100, 0.0156, 0.0313, 0.0625, 0.125, and 1.00 fg∙mL^−1^ AFB_1_. Both from Geleta et al. [72]. (**E**) Schematic illustration of magnetic beads assisted bi-nanozyme signal amplification for detection of circulating tumor cells. (**F**) DPV responses to MCF-7/aptamer/Fe_3_O_4_NPs/rGO/MoS_2_/GCE-fabricated cytosensor after capturing different concentrations of MCF-7 cells from (a) to (h): 0, 15, 20, 25, 30, 35, 40 and 45 cells∙mL^−1^ in 0.01M PBS (pH=5.0) with 0.1mM of H_2_O_2_ and 0.2mM of TMB. Both from Tian et al. [74].

Good biocompatibility of Gr/2D MoS_2_ materials has led many researchers to modify these materials with biological macromolecules such as nucleic acid aptamers, enzymes or antibodies. In case of enzymes, it was demonstrated that layered 3D structures help to increase enzymes’ stability and protect them from loss of activity. Several mediator-free enzyme-based biosensors benefiting from Gr/MoS_2_ properties were described. Among others, Yoon et al. designed the H_2_O_2_ biosensor based on myoglobin (Mb) redox activity [60]. In detail, they encapsulated MoS_2_ nanoparticles within GO and used this composite material to modify gold electrodes via chemical linker (the principle can be seen in Figure 4A). Mb is able to mediate electrochemical reduction of H_2_O_2_ due to the presence of iron within its core [61]. They compared the performance of the designed electrode with Mb/MoS_2_ and Mb/GO. Mb/GO/MoS_2_ showed an enhanced electrochemical signal even in the presence of AA, NaNO_2_ and NaHCO_3_. The amperometric read-out at −0.3 V vs. Ag/AgCl reached a LOD of 20 nM H_2_O_2_. The same approach with immobilization of Mb was used for biosensing of nitric oxide and nitrite ions [62,63]. Alternatively, hemoglobin can be used instead of Mb as showed by Liu et al. [64]. Jeong et al. compared the performance of planar Gr/MoS_2_ and Gr/MoS_2_ with 3D structure as enzymatic glucose biosensors [65]. They immobilized glucose oxidase (GOx) on glassy carbon electrodes modified with the mentioned composites and evaluated them with amperometric detection using flow-injection analysis. In general, glucose enzymatic biosensors sense H_2_O_2_ generated by oxidation of glucose by GOx. They summarized that the 3D-based biosensor possessed considerably higher sensitivity (3.36 μA∙mM^−1^) than a biosensor using the same but planar material (0.11 μA∙mM^−1^). At the H_2_O_2_ oxidation potential of −0.45 V the minimal influence of common interferences such as AA, DA or UA was observed. Graphene paper supported MoS_2_ nanocrystals monolayer with Cu submicron buds for biosensing of lactate and sensing of glucose in sweat, as reported by Wang et al. [66]. In this approach Cu buds enabled direct oxidation of glucose at +0.42 V vs. saturated calomel electrode (SCE) as was reported before [67,68]. For glucose, they reached LOD of 500 nM and linear dynamic range of 5–1775 μM with minimal influence of ions abundant in sweat such as Na^+^, Cl^−^, K^+^, Ca^2+^ and Mg^2+^. Further, they modified the mentioned electrode with lactate oxidase. Lactate oxidase is able to convert lactic acid to pyruvate and H_2_O_2,_ which enabled the indirect quantification of the concentration of lactic acid. LOD of 0.1 μM was obtained for lactate but as authors point out, low detection limits are not mandatory for sweat analysis and stability, selectivity and wide linear range are preferred. Total concentration of phenolic compounds in red wine samples based on carbon screen-printed electrode modified with graphene quantum dots, MoS_2_ and *Trametes versicolor* laccase (TvL) was reported by Vasilescu et al. (Figure 4B) [69].

Apart from nucleic acid hybridization sensors, electrochemical signal transduction is highly suitable for detection of aptamer–protein interaction. Aptamers are single-stranded nucleic acid (DNA or RNA) which possess high affinity to target molecules, comparable to or even higher than antibodies. In comparison with antibodies, which are still taken as a golden standard in biorecognition elements, aptamers are about 10-times smaller, more thermally stable and cheaper. Since aptamers are selected in vivo, their sequence can be selected to preserve desired function even in non-physiological pH or high salt concentration (important for electroanalysis). Since no animals are used for aptamer production, molecules which do not cause immune response such as toxic compounds or small molecules such as ions can be used to produce aptamers. In response to these facts, aptamers are frequently used as biorecognition elements in many different analytical applications [70]. Among others, electrochemical aptasensing is rapidly developing and covers several fields such as food safety, environmental hazards, medical diagnosis, etc.

A voltammetric lipopolysaccharides (LPS) aptasensor benefiting from advanced properties of graphene and MoS_2_ composite was reported by Yuan et al. [71]. They used large specific surface of polyethyleneimine (PEI) functionalized rGO and MoS_2_ composite (PEI–rGO–MoS_2_) as a carrier for an electrochemical label—toluidine blue (TB). More precisely, they modified GCE with PEI–rGO–MoS_2_ and loaded it with TB. Next, they used gold nanoparticles (AuNPs) to attach thiolated LPS aptamer on the electrode and used bovine serum albumin (BSA) to block the electrode against unspecified binding of LPS. In the presence of LPS in analyzed samples the TB reduction signal (−0.35 V vs. SCE) gradually decreased. The response of the aptasensor linearly decreased with logarithm of LPS concentration in the range of 5.0 × 10^−5^ ng∙mL^−1^ to 2.0 × 10^2^ ng∙mL^−1^ with the LOD of 3.01 × 10^−5^ ng∙mL^−1^. Their sensor showed good performance in the presence of common serum interferents such as BSA, AA, DA or glucose and showed recoveries in the range 101–103% in spiked serum samples. Aflatoxin B_1_ (AFB_1_) was target of the aptasensor designed by Geleta et al. [72]. They synthesized rGO, MoS_2_ and polyaniline (PANI) composite covered with chitosan (CS). GCE modified as mentioned was used to immobilize thiolated AFB_1_ aptamer via AuNPs (Figure 4C). After aptamer immobilization, the surface excessive active sites were blocked with 6-mercapto-1-hexanol. They used [Fe(CN)_6_]^3−/4−^ as an electrochemical reporter and observed a decrease of its DPV signal with increasing concentration of AFB_1_ in analyzed samples. They obtained a remarkable LOD of 0.002 fg∙mL^−1^ and a calibration curve with a linear range of 0.01 fg∙mL^−1^ to 1.0 fg∙mL^−1^ (Figure 4D).

Human papillomavirus (HPV) aptasensor was reported by Chekin et al. [73]. HPV is non-enveloped dsDNA virus that infects the epithelium and is associated with oncogenic risk. Since this virus is essential for the development of cervical cancer it is accepted as its molecular biomarker. They decided to detect HPV-16 via its L1 capsid protein. They drop-casted porous rGO on GCE and subsequently drop-casted MoS_2_ on rGO-modified GCE. GCE/rGO/MoS_2_ electrode was chemically functionalized using physisorption of thiol ligands (mixture of PEG and 11-mercaptoundecanoic acid (MUA)). NH_2_ functionalized L1 protein aptamer was subsequently immobilized on the electrode using carbodiimide chemistry (EDC/NHS). They used DPV to detect (Fe(CN)_6_)^4−^ as a mediator whose signal showed a constant decrease in the L1 protein concentration range of 0.2–2ng∙mL^−1^ and LOD 0.1–2ng∙mL^−1^. rGO/MoS_2_ nanosheets and Fe_3_O_4_ NPs nanozyme synergic catalytic activity was used to amplify the signal of a MCF-7 cytosensor [74]. They used rGO/MoS_2_ composite for electrode modification due to its high surface area, fast electron transfer and good biocompatibility. For MCF-7 cells preconcentration, they used aptamer-modified superparamagnetic Fe_3_O_4_ nanoparticles which were attracted by including attached cells to the surface of GCE via magnetic field (Figure 4E). Both materials, rGO/MoS_2_ and Fe_3_O_4_ NPs, possessed a synergetic effect in ability to reduce H_2_O_2_, and thus mediate 3,3′,5,5′-tetramethylbenzidine (TMB) oxidation (+0.3 V vs. SCE). TMB oxidation product was analyzed using DPV and the concentration of MCF-7 cells in samples was reported. They obtained a LOD of 6 cells∙mL^−1^ and a linear range over 15–45 cells∙mL^−1^ (Figure 4F).

## 4. Conclusions

In summary, graphene-based materials have attracted great interest from the scientific community. Various fabrication methods which produce composites were developed in efforts to control their structure. The research in the field of graphene-based material composites with MoS_2_ is in its starting phase and our understanding of the fundamental connection between composite structures and their properties is limited. Methods mentioned in this review bring advantages but still face several challenges and their further development is needed.

Benefits of such composite materials for electroanalytical chemistry were demonstrated in many publications and are promising for further development in this field. Firstly, graphene-based material composites with MoS_2_ possess high electrocatalytic activity, good electric conductivity and high concentrations of electroactive sites. Such properties enable direct oxidation/reduction of various electroactive compounds, bringing higher sensitivity, selectivity and better peak-to-peak separation in complex samples. Rich functionalities of composites provide possibilities of biorecognition element immobilization. Increased electrode surfaces, often with rich 3D structures enable stable and efficient immobilization of enzymes and promote the development of third generation electrochemical biosensors independent of redox mediators or oxygen. The protective environment of graphene-based material composites with MoS_2_ provides immobilized enzymes and antibodies protection from degradation and helps to retain their function. Regarding aptamers, here such biosensors take advantage of increased surface and enhance the electrochemical signal of the reporter.

Gr/MoS_2_ was shown to be a beneficial material with several attractive properties. However, the aim of all advanced materials should be application in the real world. Here, we see the possibilities for integration of Gr/MoS_2_ to currently developed platforms such as screen-printed electrodes or those currently being developed, such as promising paper-based electrodes.
